# Evolutionary Aspects of Hypericin Productivity and Endogenous Phytohormone Pools Evidenced in *Hypericum* Species In Vitro Culture Model

**DOI:** 10.3390/plants11202753

**Published:** 2022-10-18

**Authors:** Kalina Danova, Vaclav Motyka, Antoaneta Trendafilova, Petre I. Dobrev, Viktorya Ivanova, Ina Aneva

**Affiliations:** 1 Institute of Organic Chemistry with Centre of Phytochemistry, Bulgarian Academy of Sciences, Acad. Georgi Bonchev Str., bl.9, 1113 Sofia, Bulgaria; 2 Institute of Experimental Botany of the Czech Academy of Sciences, Rozvojová 263, 165 02 Prague, Czech Republic; 3 Institute of Biodiversity and Ecosystem Research, Bulgarian Academy of Sciences, 2 Gagarin Str., 1113 Sofia, Bulgaria

**Keywords:** *Hypericum* evolution, in vitro culture, wild habitats, hypericin, endogenous phytohormones

## Abstract

Shoot cultures of hypericin non-producing *H. calycinum* L. (primitive *Ascyreia* section), hypericin-producing *H. perforatum* L., *H. tetrapterum* Fries (section *Hypericum*) and *H. richeri* Vill. (the evolutionarily most advanced section *Drosocarpium* in our study) were developed and investigated for their growth, development, hypericin content and endogenous phytohormone levels. Hypericins in wild-growing *H. richeri* significantly exceeded those in *H. perforatum* and *H. tetrapterum*. *H. richeri* also had the highest hypericin productivity in vitro in medium supplemented with 0.2 mg/L *N*^6^-benzyladenine and 0.1 mg/L indole-3-butyric acid and *H. tetrapterum*—the lowest one in all media modifications. In shoot culture conditions, the evolutionarily oldest *H. calycinum* had the highest content of salicylic acid and total jasmonates in some of its treatments, as well as dominance of the storage form of abscisic acid (ABA-glucose ester) and lowest cytokinin ribosides and cytokinin *O-*glucosides as compared with the other three species. In addition, the evolutionarily youngest *H. richeri* was characterized by the highest total amount of cytokinin ribosides. Thus, both evolutionary development and the hypericin production capacity seemed to interact closely with the physiological parameters of the plant organism, such as endogenous phytohormones, leading to the possible hypothesis that hypericin productivity may have arisen in the evolution of *Hypericum* as a means to adapt to environmental changes.

## 1. Introduction

*Hypericum perforatum* L. is one of the most widely used and studied medicinal plant species, with origins dating back to the first century AD [1]. The pharmacologically used aerial parts of the plant (*Herba hyperici*) are characterized by containing a remarkably broad spectrum of phytochemical constituents such as polyphenolic compounds, flavonoids, naphthodianthrones, phloroglucinols and terpenes [2,3,4]. This is the prerequisite for the wide range of pharmacological activities characteristic of preparations of this species, such as antidepressive, antitumor, antiviral and antibiotic [5,6,7]. In addition to the well-known *H. perforatum* L., the genus *Hypericum* includes over 484 species distributed worldwide and classified into 36 sections. Either naturally occurring or introduced *Hypericum* species are found on all continents of the world except Antarctica, with habitus ranging from trees to herbaceous plants [8]. 

Hypericin and pseudohypericin are condensed naphthodianthrones found only in the genus *Hypericum.* They were first isolated from *H. perforatum* about 80 years ago, and this species has been the main source of their supply ever since ([9] and references cited therein). Hypericin is considered one of the most important bioactive constituents of *Herba Hyperici*. Its remarkable physicochemical properties and biological activity are due to its unique chemical structure [9]. Hypericin is considered one of the most potent photosensitizers in nature, a phenomenon that determines the active exploration of its potential in photodynamic cancer therapy [10]. In addition to exploiting the cytotoxic effect of hypericin on cancer cells as a result of its photoactivation, interesting results have also been obtained regarding the biological activity of non-photoactivated hypericin. For example, non-photoactivated hypericin has been shown to affect, albeit to a lesser extent than its photoactivated form, key steps of angiogenesis in a model of bovine vascular endothelial cells [11]. In addition, non-photoactivated hypericin has also been shown to possess anti-metastatic properties in an in vivo model [12]. Hypericin has shown selectivity towards oncogenic tissues compared to non-pathologic ones. For example, the accumulation of hypericin in primary and metastatic lung tumors was twice that in healthy lung tissue [12]. Hypericin has also been shown to possess antiviral and antidepressant properties [13]. The strong antiviral activity of hypericin has been demonstrated against non-enveloped RNA viruses such as human immunodeficiency virus (HIV) and hepatitis C virus (HCV), as well *Coronaviridae* viruses such as γ-CoV infectious bronchitis virus and α-CoV ([13] and references cited therein). Molecular docking experiments have also demonstrated the antiviral potential of hypericin in SARS-CoV-2 by targeting the 3CL protease [14,15,16].

Many factors are thought to be responsible for the phytochemical variability of *H. perforatum*, such as genotype, geographical origin, harvest stage and plant age [17]. Moreover, intragenetic studies have shown that hypericin content is related to the distribution of sections and evolutionary development of representatives of the genus *Hypericum* [18]. Thus, phytochemical research has shown that some *Hypericum* representatives, such as the wild *H. boissieri*, *H. barbatum* and *H. rumeliacum* (section *Drosocarpium*) are able to accumulate two- to fourfold higher amounts of hypericins than *H. perforatum* (the older *Hypericum* section) [18,19,20]. 

The Bulgarian flora offers a wealth of wild accessions of 22 *Hypericum* species. Of these, five are Balkan and one is a Bulgarian endemic species (but the latter, *H. setiferum Stef.*, section *Drosocarpium*, is extinct) [21]. Of these, one of the representatives belongs to the most primitive section *III. Ascyreia* Choisy (the hypericin-non-producing *H. calycinum* L. studied in the present work), one to the section *V. Androsaemum* (Duhamel) Godron, four to the section *IX. Hypericum* (including *H. perforatum* L. and *H. tetrapterum* Fries. investigated in the present work), one to the section *X. Olympia* (Spach) Nyman, one to the section *XI. Campylopus* Boiss., eight to the section *XIII. Drosocarpium* Spach (including *H. richeri* Vill. studied in the present work), one to the section *XIV. Oligostema* (Boiss.) Stef., one to the section *XV. Thasia* Boiss., one to the section *XVII. Hirtella* Stef., two to the section *XVIII. Taeniocarpium* Jaub. et Spach. and one to the section *XXVII Adenosepalum* Spach. [18,21,22,23]. 

Plant cell tissue and organ culture is a practical and flexible tool for elucidating processes within the plant organism related to the production of secondary metabolites. In our previous work on shoot cultures of *H. richeri, H. perforatum, H. tetrapterum* and *H. calycinum,* it was found that three groups can be distinguished in terms of endogenous total cytokinin (CK) pools [24]. Thus, the highest values for this parameter were observed in the evolutionarily most advanced *H. richeri*, then intermediate values in *H. perforatum* and *H. tetrapterum*, and the lowest total CK pools in *H. calycinum*. Similar dependencies were observed for the levels of *trans*-zeatin (*t*Z)-type CKs. In contrast, *cis-*zeatin (*c*Z)-types dominated in *H. tetrapterum*, followed by *H. perforatum* and *H. richeri.* However, the *c*Z-type CKs again showed a clear minimum in the evolutionarily older *H. calycinum* [24]. 

**Figure 1:**
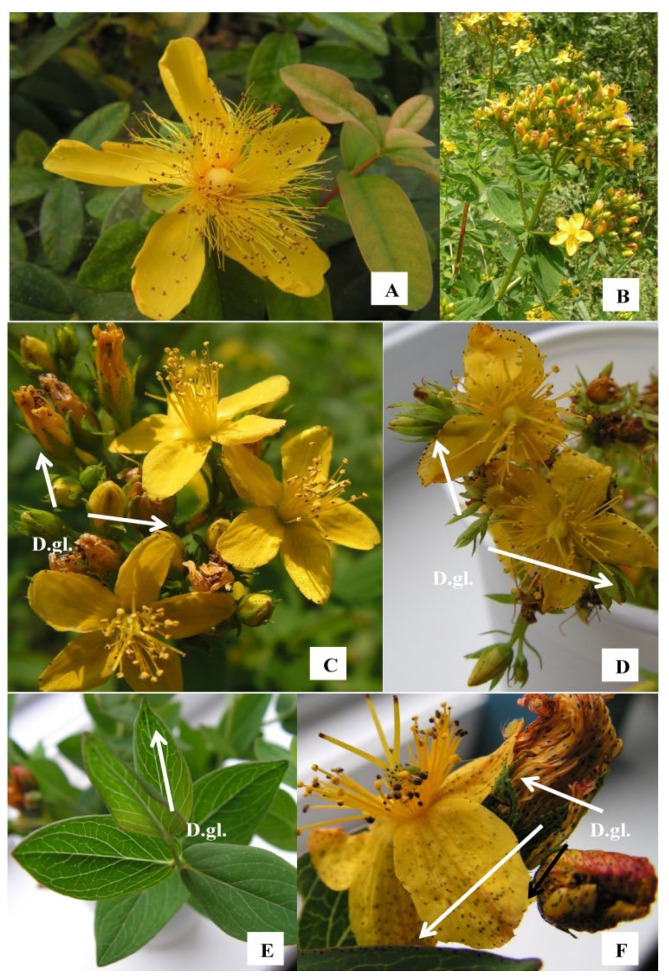
In situ growing *H. calycinum* L. (**A**); *H. tetrapterum* Fries. whole plant habitus (**B**) and blossoms with visible dark glands (D.gl.) on petal and sepal ribs (**C**); *H. perforatum* L. blossoms with dark glans (**D**); *H. richeri* Vill. leaves with scarce dark glands visible on leaves margins of non-blossoming aerial parts (**E**); and *H. richeri* Vill. blossoms with abundant dark glands visible on petals and sepals, as well as leaf ribs in proximity of blossoms (**F**).

The present work was motivated by the comparison of the phytochemical potential of the widely studied *H. perforatum* with that of other representatives of the genus belonging to sections with different evolutionary stages, and related the observed dependencies to the physiological status of the plant under tissue culture conditions. Particular attention was paid to further investigating the changes in the pools of some phytohormones and relating them to hypericin productivity under different in vitro conditions. For this purpose, the above parameters were determined for the hypericin-non-producing *H. calycinum* L. (the most primitive *Ascyrea* section in the present work), the hypericin-producing *H. perforatum* L., *H. tetrapterum* Fries. (section *Hypericum*) and the evolutionarily most advanced *H. richeri* Vill. (section *Drosocarpium*) (Figure 1).

## 2. Results and Discussion

### 2.1. Effect of Plant Growth Regulators on In Vitro Culture Development

**Figure 2:**
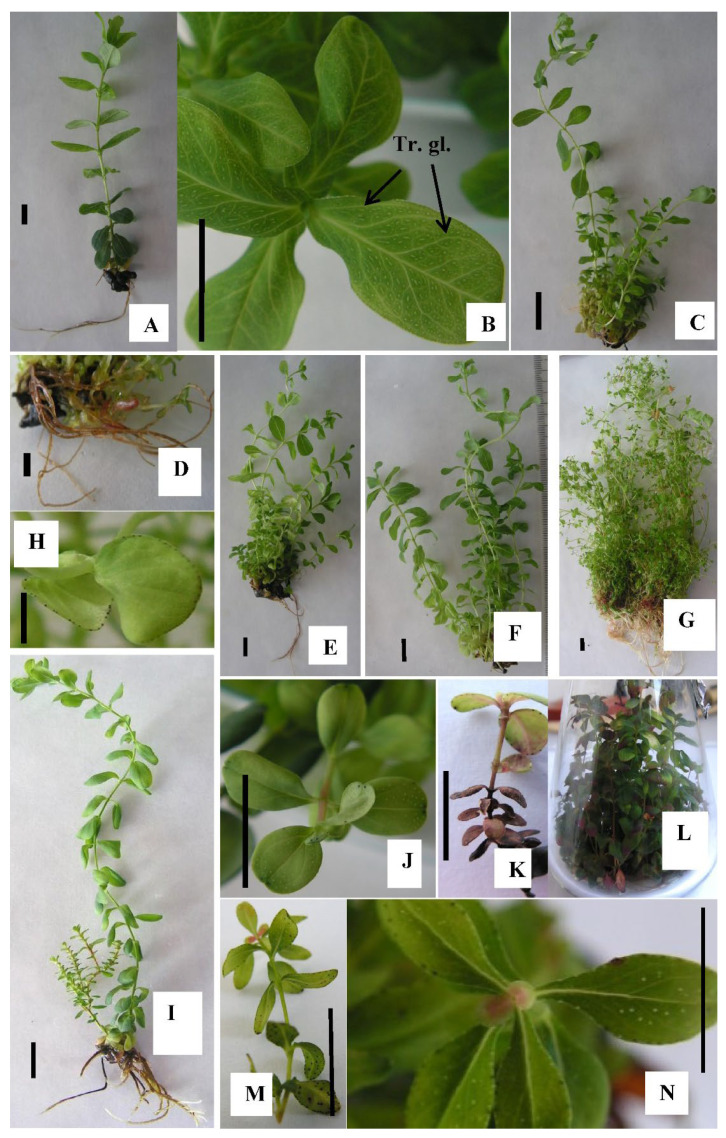
In vitro cultivated shoot cultures of *H. calycinum* L. in M_0 culture medium (**A**) with well visible translucent glands (Tr. gl.) on the leaves (**B**); stimulation of axillary shoot formation (**C**) and rooting (**D**) in M_1 medium; stimulation of the height of auxillary shoots formed in M_2 medium (**E**); further stimulation of the height of axillary shoots and inhibition of rooting in M_3 medium (**F**); intensive biomass formation (**G**) and dark glands on leaves margins (**H**) in in PGR-free M_0 medium *H. tetrapterum* Fries.; biomass formation (**I**) and dark and translucent glands on leaves (**J**) in *H. perforatum* L. in M_0 medium; growth inhibition and necrosis in PGR-free M_0 medium in *H. richeri* Vill. (**K**); biomass formation stimulation *H. richeri* Vill. (**L**) with well visible translucent and dark glands on the leaves (**M**,**N**) in M_2 medium. Space bar = 1 cm.

The translucent glands characteristic of essential oil and phloroglucinol accumulation in hypericin-non-producing *Hypericum* species were clearly visible in *H. calycinum* (Figure 1 and Figure 2B), whereas the dark hypericin glands associated with hypericin accumulation were evident in the other three *Hypericum* species (see Figure 1 and Figure 2H,J,K,M,N). Cultivation of *H. richeri* for a period longer than 4 weeks in medium without plant growth regulators (PGRs) was not possible, because no multiplication occurred and explants gradually decayed during the cultivation period of the entire experiment (Figure 2K). The addition of PGRs strongly stimulated biomass formation compared to the PGR-free control in all four species of *Hypericum* shoot cultures. 

**Figure 3:**
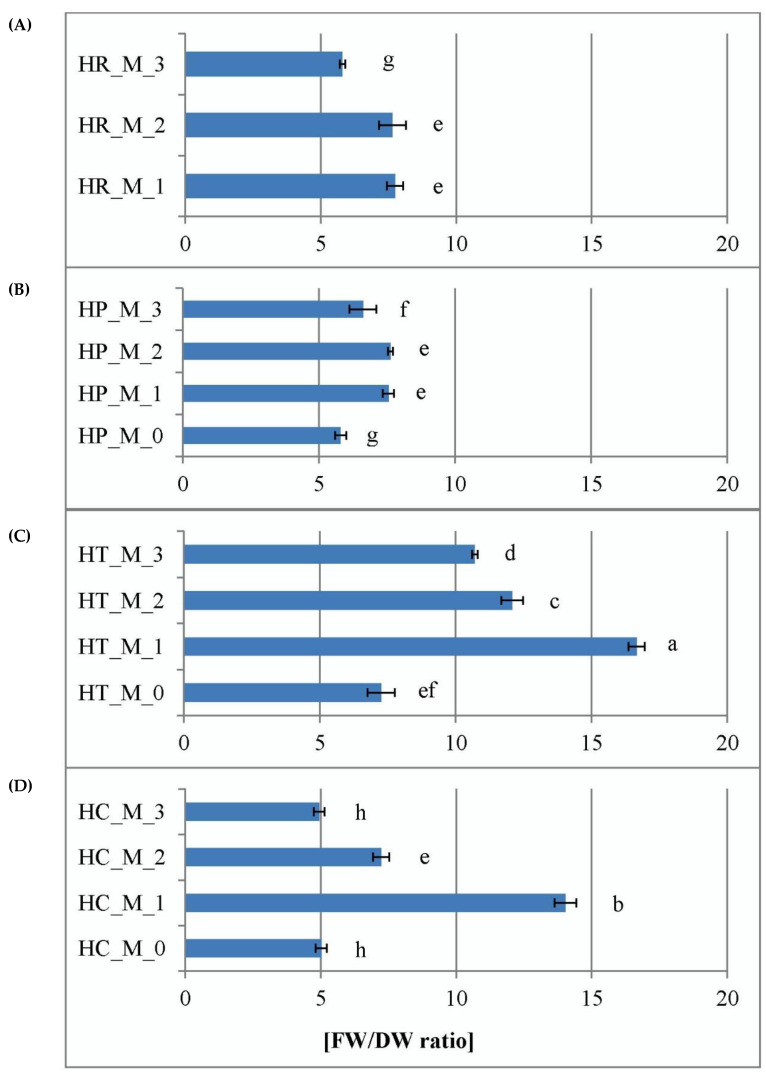
Effect of plant growth regulators on tissue hydricity in *Hypericum* ssp. studied in vitro. M_0—PGR-free (**A**), M_1—0.2 mg/L BA (**B**), M_2—0.2 mg/L BA + 0.1 mg/L IBA (**C**) and M_3—0.1 mg/L BA + 0.2 mg/L IBA (**D**) supplemented media, HR—*H. richeri* Vill., HP—*H. perforatum* L., HT—*H. tetrapterum* Fries. and HC—*H. calycinum* L. Same letters denote statistically non-significant differences and different letters denote statistically significant differences in mean values when comparing the parameter between all samples.

Estimation of the FW/DW ratio of the samples as a result of the different treatments was used as a simple physical characterization of the extent of water retention in plant tissues. *H. calycinum* showed the lowest tissue hydricity in M_0 (PGR-free) and M_3 (0.1 mg/L BA + 0.2 mg/L IBA) treatments compared with the other three *Hypericum* species (Figure 3). The highest plant tissue hydration for all PGR-treated samples was generally observed in *H. tetrapterum* compared to the other three species (Figure 3). *H. richeri* and *H. perforatum* showed a comparable ability to accumulate water in plant tissues, with the lowest values for this parameter observed in the M_3 treatment of *H. richeri*. Water retention in plant tissues is an important physiological parameter in plant cell tissue and organ culture development. Foliar water content (estimated as the difference between fresh and dry leaf weights) does not proportionally relate to the leaf dry weight during growth, and has been shown to play an important role in photosynthesis. Studies have shown that dependencies obtained upon scaling between fresh and dry weight are species specific [25]. This feature was also confirmed by our experimental results outlining *H. calycinum* as the species with the lowest and *H. tetrapterum* with the highest water retention capacity of the plant tissues.

The morphological observations of the translucent and dark glands in the four *Hypericum* in vitro models in the present work confirm the literature data on their morphological structures and relationship with the secondary metabolites produced. Previous studies have shown that the phloroglucinol hyperforin is biosynthesized in the translucent glands and their surrounding cells, with its isoprenoid moiety synthesized via the biogenetic pathway of monoterpenoids, sharing the same constituents with the essential oils also present in the translucent glands [26,27]. The site of hyperforin biogenesis is thought to be the chloroplasts of the cells surrounding the translucent glands [28].

Although it is widely accepted that hypericin accumulation is undoubtedly related to dark glands in *Hypericum* tissues [9,20], its biosynthetic pathway is still unknown. A recent work by Pradeep and Franklin [29] shed more light on the matter by conducting an experiment to reduce the number and size of dark glands in *H. perforatum* shoot cultures by treatment with a sub-lethal concentration of glyphosate. As a result, the accumulation of hypericins and their precursors (emodin and bisanthrones) was significantly inhibited, and this process was reversible after glyphosate was removed from the medium. Polyketide synthases and phenolic oxidative coupling proteins were downregulated after treatment and upregulated again after glyphosate removal. Interestingly, phenolic compounds were largely unaffected by treatment.

### 2.2. Total Hypericins Content of Field and In Vitro Collected Samples

**Figure 4:**
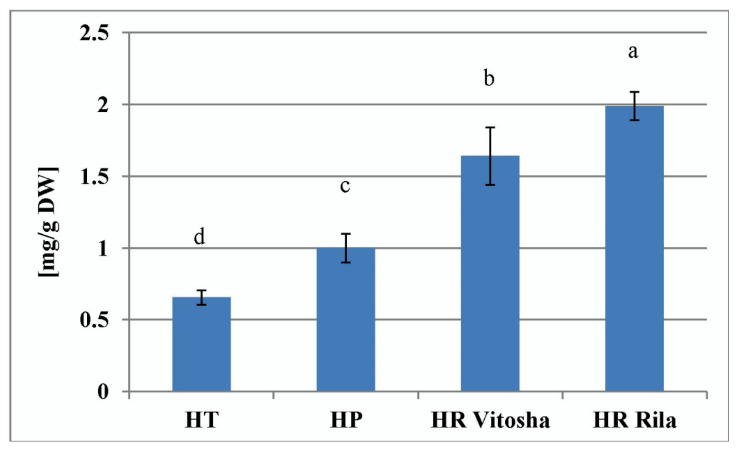
Total hypericin content of wild growing *Hypericum* ssp. samples. HT—*H. tetrapterum* Fries., HP—*H. perforatum* L., HR Vitosha—*H. richeri* Vill. from Vitosha Mountain accession, HR Rila—*H. richeri* Vill. from Rila Mountain accession. All measurements were performed in triplicate and the values are given as mean ± SD. Different letters denote statistically significant differences of the means.

Wild collections of *H. richeri* from both Vitosha and Rila mountain wild accessions proved superior to the other two *Hypericum* species in terms of total hypericin production. The lowest hypericin productivity was found for *H. tetrapterum* (Figure 4).

**Figure 5:**
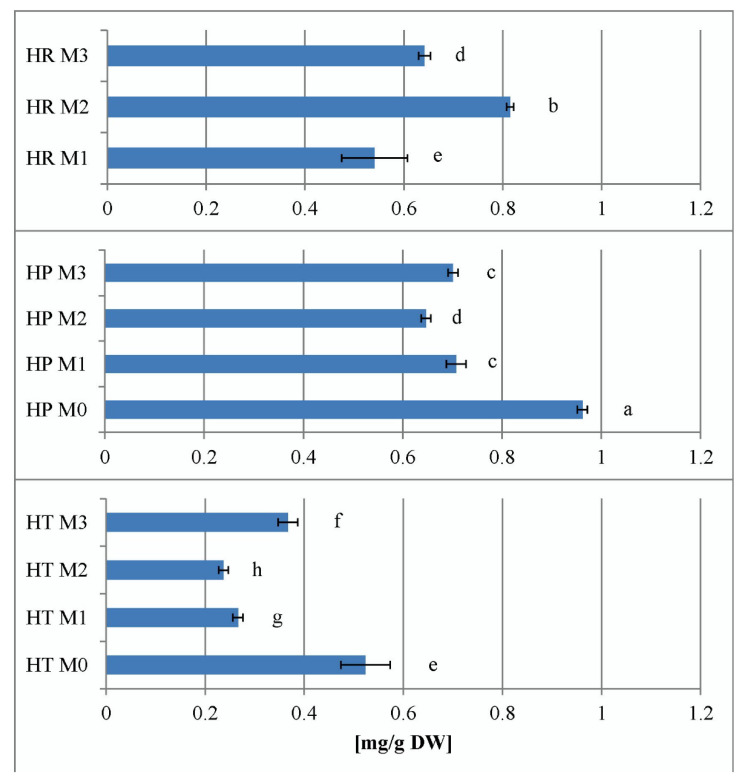
Total hypericin content in the three *Hypericum* ssp. in M_0—PGR-free, M_1—0.2 mg/L BA, M_2—0.2 mg/L BA + 0.1 mg/L IBA and M_3—0.1 mg/L BA + 0.2 mg/L IBA supplemented media. HP—*H. perforatum* L., HR—*H. richeri* Vill. and HT—*H. tetrapterum* Fries. All measurements were performed in triplicate and the values are given as mean ± SD. Same letters denote non-significant differences and different letters denote statistically significant differences of the means.

When considering the total hypericins content of *H. perforatum* and *H. tetrapterum* in vitro, it became clear that in both species, PGR supplements decreased the total hypericins content so that M_0 treatment proved to be optimal for in vitro hypericin production. In general, *H. tetrapterum* was found to have the lowest in vitro hypericin production potential of all three hypericin-producing species. For *H. richeri*, the intragenic comparison could only be based on the M_1, M_2 and M_3 treatments because no M_0 sample was available. When comparing the same PGR treatments of the three species, the highest hypericin production was found in H. richeri in the sample M_2 (0.2 mg/L BA + 0.1 mg/L IBA) (Figure 5). 

Plant cell tissue and organ cultures of *Hypericum* species have been intensively studied in the literature from 1992 with the pioneering work of Zdunek and Alfermann [30] and Čellarova et al. [31] to date. Various in vitro model systems have been investigated, with the vast majority of literature reports on the production of secondary metabolites in tissue cultures focusing primarily on *H. perforatum* ([1] and references cited therein). Important tools for improving biotechnological hypericin production have been the multiple approaches to biotic and abiotic elicitation in various models of *Hypericum* plant cell tissue and organ culture ([32] and references cited therein). Research was also conducted on *Hypericum* species from other sections of the genus, although on a much smaller scale. The effects of various elicitors, as well as PGRs, on the production of secondary metabolites of *H. hirsutum* (section *XVIII. Taeniocarpium*) and *H. maculatum* (section *IX. Hypericum*) were studied, and showed that salicylic acid (SA) increased the production of hypericin (7.98-fold) and pseudohypericin (13.58-fold) in *H. hirsutum* at 50 μM and at 200 μM increased the production of hypericin (2.2-fold) and pseudohypericin (3.94-fold) in *H. maculatum* [33]. By applying the processes of de-differentiation in vitro, valuable secondary metabolites of lower molecular weight have been obtained. Thus, the callus cultures of *H. perforatum* have proved to be a promising new source for the production of secondary metabolites with xanthone structure [34]. The potential of representatives of the more highly developed *Hypericum* sections to produce high amounts of naphthodianthrone derivatives has been discussed in the literature. For example, in a comparative work with 36 *Hypericum* species, Kitanov [18] found the highest hypericin content in taxa of the sections *Drosocarpium*, *Hypericum*, and *Thasia*, which were the most evolutionarily advanced in the study. It was concluded that hypericin and pseudohypericin play an important role in the intragenic classification of *Hypericum*, being characteristic of the phylogenetically advanced sections (*IX–XV*, *XXVII*) and absent in representatives of the primitive sections (*III*, *V–VIII*) of the genus. However, the role of plant origin and environmental factors also appears to correlate with hypericin production. Thus, in a comparative analysis of five samples of *H. perforatum* (section *IX. Hypericum*) collected from different geographical locations with four *Hypericum* species native to the Balkan Peninsula (*H. maculatum* subsp. *immaculatum*—section *IX. Hypericum*, *H. olympicum*—section *X. Olympia*, *H. richeri* subsp. *grisebachii* and *H. barbatum*—both section *XIII. Drosocarpium*), Božin et al. [35] found that samples of *H. perforatum* showed remarkable variability depending on their origin, with the highest hypericin concentration among the probes being comparable to that of *H. barbatum*. The samples of *H. olympicum* had lower hypericin concentrations, and interestingly, no hypericin was detected in the accessions of *H. maculatum* and *H. richeri*, studied in the latter work. 

The hypericin values determined in the present work for the samples of the wild-collected *Hypericum* species confirm the above literature data and show that *H. richeri*, which belongs to the most advanced *Drosocarpium* section, has the highest capacity. The lowest hypericin levels were found for *H. tetrapterum* in both *in situ* and in vitro samples. When comparing the PGR-treated samples of the *Hypericum* species producing hypericin, the in vitro capacity of *H. richeri* to produce the highest levels of hypericin was found for the M_2 treatment. 

### 2.3. Effect of Plant Growth Regulators Treatments on Endogenous Phytohormone Levels In Vitro 

#### 2.3.1. Levels of Abscisic Acid and Its Conjugates In Vitro

**Figure 6:**
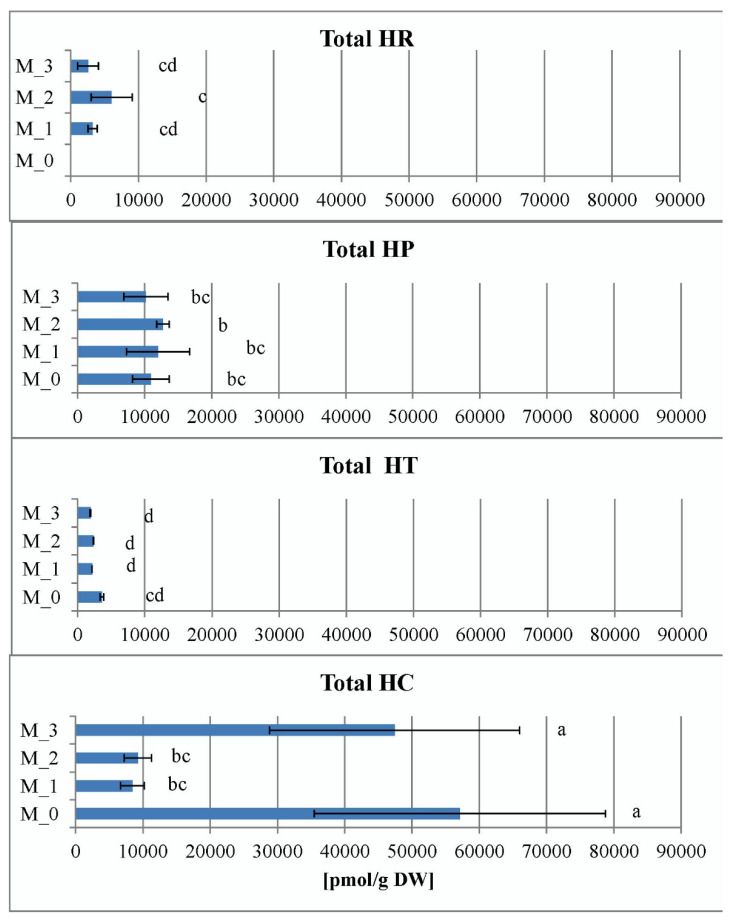
Representation of the total sum of the two abscisic acid (ABA) forms: ABA and its storage conjugate ABA–glucose ester (ABA–GE) in the four *Hypericum* species in M_0—PGR-free, M_1—0.2 mg/L BA, M_2—0.2 mg/L BA + 0.1 mg/L IBA and M_3—0.1 mg/L BA + 0.2 mg/L IBA supplemented media, HR—*H. richeri* Vill., HP—*H. perforatum* L., HT—*H. tetrapterum* Fries. and HC—*H. calycinum.* Same letters indicate statistically non-significant differences in mean values when comparing the parameter between all samples.

As a general observation (with the exception of M_0 and M_3 *H. calycinum* treatments) the total sum of abscisic acid (ABA) and its glucose ester (ABA-GE) did not show much variation within the same species as a result of the applied PGR treatments (Figure 6). Apart from the exceptions mentioned above, the overall lowest values were recorded for *H. tetrapterum* (1906–3579 pmol/g DW) and H. richeri (2557–6034 pmol/g DW) and the highest for *H. perforatum* (10,158–12,708 pmol/g DW) and *H. calycinum* (8444–57,096 pmol/g DW) (Figure 6). Thus, the total concentration of ABA conjugates appears to be similar for *H. tetrapetrum* and *H. richeri* (which have a lower accumulation capacity) on the one hand, and *H. perforatum* and *H. calycinum* (which have a higher accumulation capacity) on the other. 

A tendency toward general analogic representation between *H. perforatum* and *H. calycinum* on one side, and *H. richeri* and *H. tetrapterum* on the other was also noted in the allocation of the two ABA forms (Figure S1). In the most primitive species, *H. calycinum*, the proportion of ABA–GE ranged from 62% to 83% in the different treatments, and ABA–GE was the dominant conjugate of ABA in all PGR treatments examined. In *H. perforatum*, the proportion of ABA–GE ranged from 41% to 54%, indicating a tendency toward equilibrium between the two forms studied in the present experiment.

Interestingly, ABA–GE was not detected at all in four *H. tetrapterum* treatments (Figure S1). In *H. richeri*, ABA–GE was observed only in the M_2 treatment, where it accounted for about one-third of the total ABA sum. 

The widely accepted concept of the role of ABA in plants is the response to biotic and abiotic stresses such as salt, cold, heat, drought and wounding, as well as exposure to pathogens and the regulation of various physiological processes in the absence of stress, such as dormancy, heterophylly, pollination and leaf senescence ([36] and references cited therein). The role of ABA in regulating plant cell turgor is expressed by prevention of opening of closed stomata and by closing open stomata, as illustrated by a model of a diurnal monitoring of ABA levels (peak at the beginning of the dark phase to ensure stomata closure and rehydration during the night, and a sharp decrease after the beginning of the light period) [37]. 

ABA levels in plants have been shown to fluctuate constantly due to its production, inactivation, and transport pathways in response to different developmental stages or to changes occurring in the environment [38]. Studies have shown that ABA is predominantly synthesized de novo in higher plants via the carotenoid pathway. ABA–GE is considered to be an inactivation end product of ABA metabolism, and the majority of this conjugate remains at constant levels in vacuoles and does not decrease in response to stress [39,40]. On the other hand, an increase in the concentration of ABA–GE was detected in the xylem of stressed plants, suggesting that it may also act as a potential stress signal from roots to shoots [41]. 

**Figure 7:**
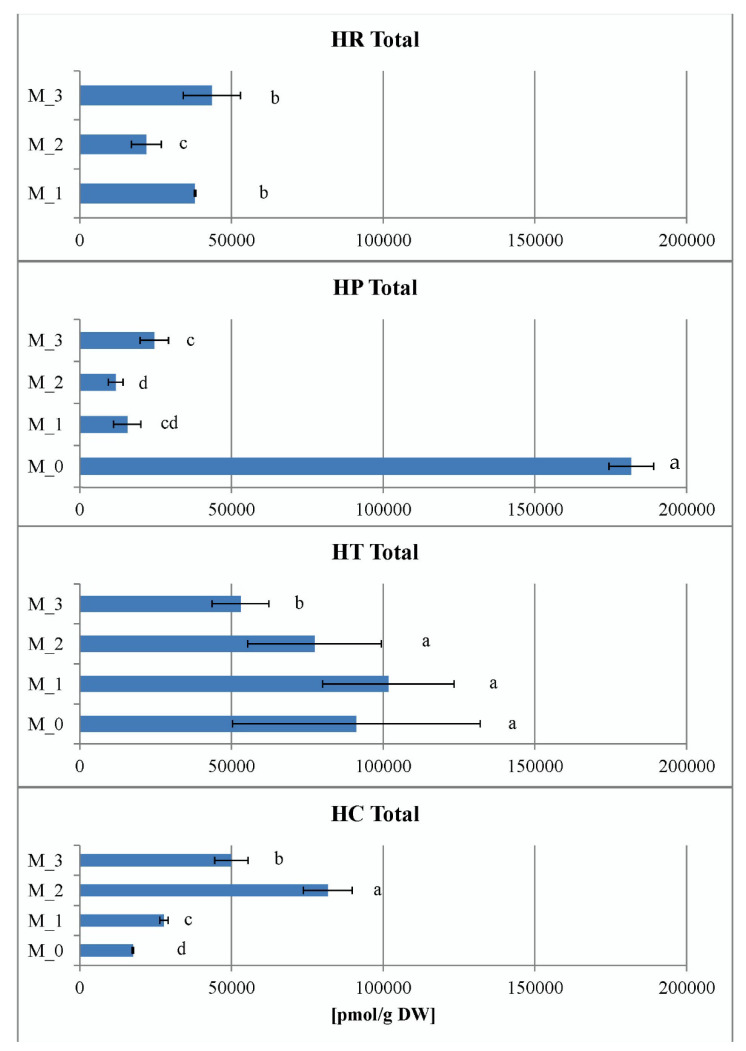
Total sum of salycilic acid (SA) and benzoic acid (BA) in the four *Hypericum* species in M_0—PGR-free, M_1—0.2 mg/L BA, M_2—0.2 mg/L BA + 0.1 mg/L IBA and M_3—0.1 mg/L BA + 0.2 mg/L IBA supplemented media. HR–*H. richeri* Vill., HP–*H. perforatum* L., HT–*H. tetrapterum* Fries. and HC–*H. calycinum*. Same letters indicate statistically non-significant and different letters indicate statistically significant differences in mean values when comparing the parameter between all samples.

In the present experiment, for the lowest hypericin producing *H. tetrapterum* there was a relation between its highest tissue water retention of all four *Hypericum* species (its physical estimation expressed as the highest FW/DW ratio, Figure 3) and the lowest total concentrations of ABA conjugates (Figure 6). Moreover, no detectable ABA inactivation process was observed in this species (a lack of ABA–GE detection in HT, Figure 7). These results could indicate intensive physiologically-active ABA mobilization in the process of water retention. In contrast, in *H. calycinum*, which has the lowest FW/DW ratios of all four *Hypericum* species (Figure 3), was found to have higher total ABA pools (Figure 6) and a clear ABA–GE dominance (Figure S1), suggesting a less intense ABA mobilization and a process of ongoing ABA inactivation. In the other two *Hypericum* species, characterized by a higher hypericin productivity, the experiment showed a clear similarity in tissue water retention capacity (Figure 3), which, however, did not correlate with the dynamics of ABA and its inactivation form (ABA–GE) as was the case for HT and HC. Thus, as far as ABA is concerned, hypericin production capacity rather than evolutionary development may be involved in the processes regulating water retention in plant tissues. 

#### 2.3.2. Levels of Benzoic and Salicylic Acid In Vitro

Phenolic compounds, BzA and SA, were found in relatively high amounts in the shoot cultures of four *Hypericum* species. Their total concentrations ranged from 11,823 to 181,761 pmol/g DW (*H. perforatum* at M_2 and M_0, respectively) (Figure 7). Observation of total SA and BzA concentrations within species and treatments showed that *H. tetrapterum* had the least variability as a result of PGR treatments compared with the other three species (Figure 7). In contrast, the highest variability was observed in *H. perforatum*, with the PGR-free control significantly exceeding the PGR treatments. In general, this parameter does not appear to be related to the evolutionary stage or hypericin productivity of the samples studied.

Further elaboration of the individual contents of BzA and SA within the species/PGR treatments showed a significant dominance of BzA over SA in all samples (Figure S2). The concentration of BzA significantly exceeded that of SA, and the highest BzA levels (99% of the total amount) were measured in *H. perforatum* at M_0 (179,303 pmol/g DW). *H. perforatum* exhibited the highest values of SA stimulation as a result of the PGR treatments (from 1% of the total amount at M_0 up to 35% at M_2) compared to the other three *Hypericum* species. The evolutionarily oldest *H. calycinum* had the highest proportion of SA in the PGR-free control M_0 (12% of the total amount) compared with the other *Hypericum* species. The plant phenolic hormone SA, derived from the building blocks of BzA, is produced in plastids and transported to the cytosol. Thus, it is an important endogenous signal for coping with environmental stress as well as for controlling vital processes such as flowering and senescence [42]. The process of SA biogenesis appeared to be strongly stimulated by PGR treatments in the hypericin-producing *Hypericum* species in the present experiment and constitutively high in the PGR-free control in the most primitive *H. calycinum*. 

#### 2.3.3. Endogenous Levels of Jasmonates In Vitro

Jasmonates are known to influence many physiological processes of plant growth and development, and especially mediating plant responses to biotic and abiotic stresses [43,44]. They have been extensively used for elicitation studies within in vitro cultures [45], and their effect on the production of secondary metabolites in culture systems has been widely described ([46] and references cited therein). 

**Figure 8:**
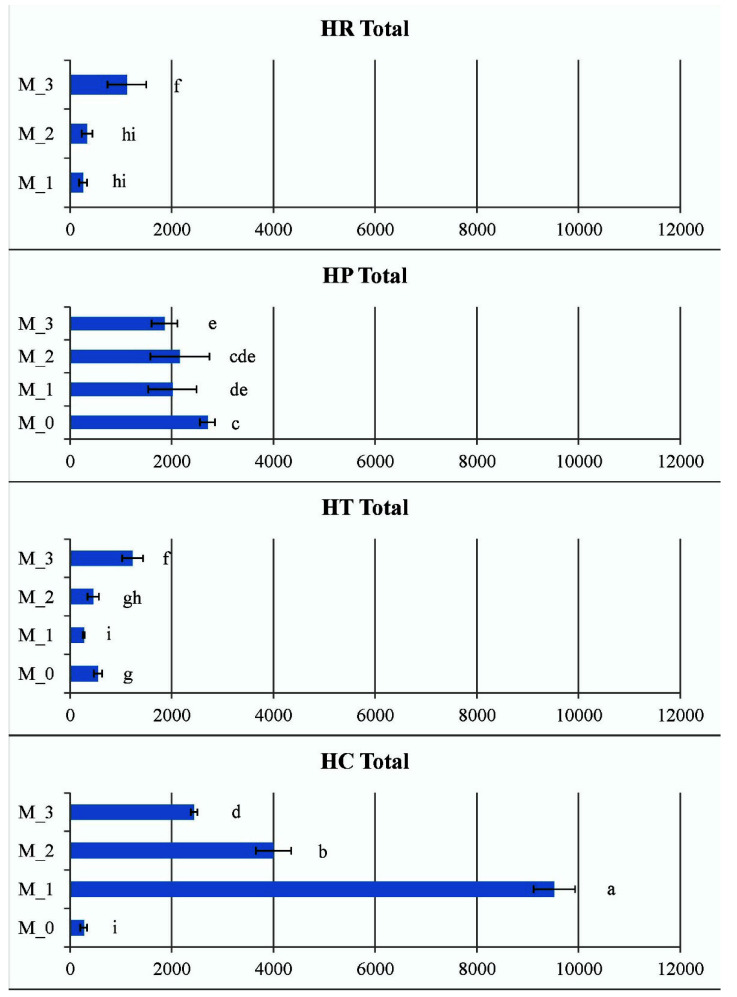
The sum of jasmonic acid (JA) and its bioactive conjugate JA-isoleucine in the four *Hypericum* species in M_0—PGR-free, M_1—0.2 mg/L BA, M_2—0.2 mg/L BA + 0.1 mg/L IBA and M_3—0.1 mg/L BA + 0.2 mg/L IBA supplemented media. HR–*H. richeri* Vill., HP–*H. perforatum* L., HT–*H. tetrapterum* Fries. and HC–*H. calycinum*. Same letters indicate statistically non-significant and different letters indicate statistically significant differences in mean values when comparing the parameter between all samples.

In the present work, the total pool of jasmonates ranged from 258 pmol/g DW in *H. richeri* (M_1) to 9521 pmol/g DW in *H. calycinum* (M_1) (Figure 8). It consisted of jasmonic acid (JA) and its conjugate JA-isoleucine (JA-Ileu), which, however, was much less abundant or even absent (Figure 8). JA-Ileu is known to be a bioactive JA metabolite that is recognized by the jasmonate receptor [47] and its formation thus represents an important step in JA signaling.

When analyzing the data obtained for total jasmonates, i.e., the sum of JA and JA-Ileu, the greatest variability was observed when comparing the PGR-free and PGR-treated samples in *H. calycinum*, as the treatments significantly increased the values of the studied parameter (Figure 8). The jasmonate levels of the PGR-treated *H. calycinum* samples exceeded those of the other three *Hypericum* species. The lowest variability between treatments was observed in *H. perforatum*, which displayed intermediate values of total jasmonates. *H. richeri* and *H. tetrapterum* had the lowest values for this parameter (Figure 8).

Analysis of the ratios between JA and JA-Ileu showed that in all samples the levels of JA significantly exceeded those of the bioactive JA conjugate (Figure S3). Although there were no substantial differences in the proportion of JA-Ileu between PGR treatments, it appears to be greatest in *H. tetrapetrum* and *H. calycinum* (up to 10 and 11%, respectively) and smallest in *H. perforatum* (max. 7%).

#### 2.3.4. Contents of Endogenous Cytokinins In Vitro

In our previous work, we determined the total amount of endogenous CKs and compared the pools and ratios between *c*Z- and *t*Z-types in *Hypericum* in vitro cultures [24]. We detected the highest pool of total CKs in the evolutionarily most advanced *H. richeri*, while the lowest amounts of total CKs, as well as *c*Z- and *t*Z-types, were found in the most primitive *H. calycinum* [24]. Therefore, we aimed here to investigate other metabolic forms of CKs (ribosides, *N*- and *O*-glucosides and methylthioderivatives) in the described *Hypericum* in vitro experimental model.

**Figure 9:**
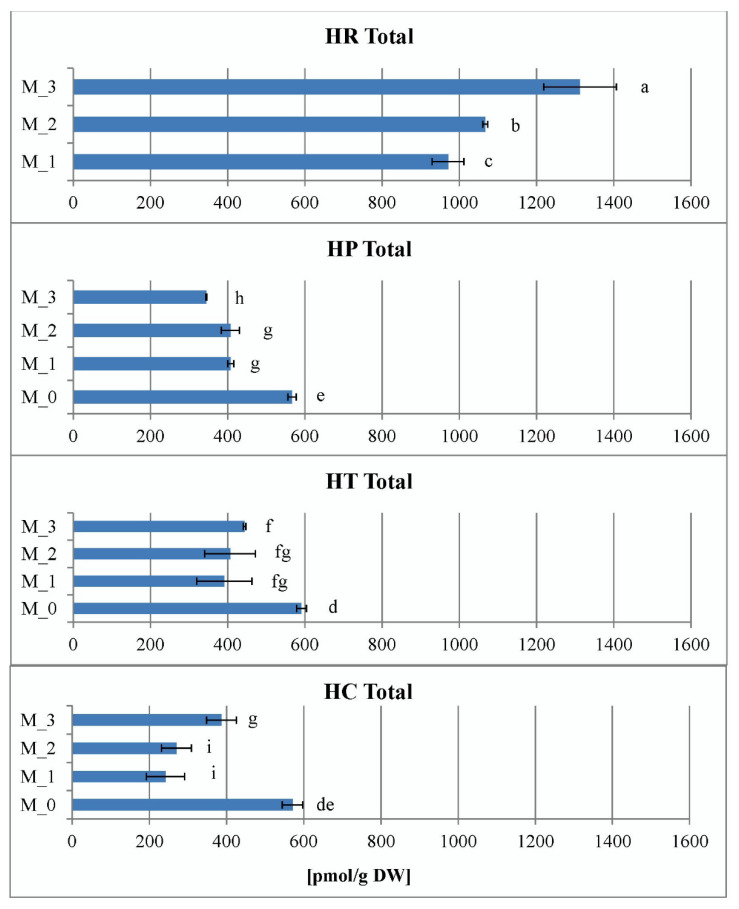
Total sum of cytokinin ribosides in the four *Hypericum* species in M_0—PGR-free, M_1—0.2 mg/L BA, M_2—0.2 mg/L BA + 0.1 mg/L IBA and M_3—0.1 mg/L BA + 0.2 mg/L IBA supplemented media, HR–*H. richeri* Vill., HP–*H. perforatum* L., HT–*H. tetrapterum* Fries. and HC–*H. calycinum*. Same letters indicate statistically non-significant and different letters indicate statistically significant differences of the mean values when comparing the parameter between all samples.

The data of total CK ribosides in vitro shown in Figure 9 represent the sums of *trans*-zeatin riboside (*t*ZR), dihydrozeatin riboside (DZR), *cis*-zeatin riboside (*c*ZR), *N^6^*-(∆*^2^*-isopentenyl)adenosine (iPR) and 2-methylthio zeatin riboside (MeSZR) analyzed in this work. The content of total CK ribosides in *Hypericum* in vitro cultures varied from 242 pmol/g DW in *H. calycinum* (M_1) to 1313 pmol/g DW in *H. richeri* (M_3). In general, it was observed in all *Hypericum* species that PGR treatment reduced the content of total CK ribosides compared to the PGR-free control (data for the PGR-free control of *H. richeri* are not available because the species cannot be maintained long-term in PGR-free medium). Intragenic comparison of the three PGR treatments showed that *H. richeri* had the highest values of the studied parameter for all treatments compared to the other three species (Figure 9). In contrast, the lowest determined values of the total CK ribosides in the experiment were found in the PGR treatments of *H. calycinum*.

When the profiles of the individual CK ribosides were elaborated, it was found that MeSZR was the most abundant metabolite in all four *Hypericum* species (Figure S4). In general, PGR treatments increased the content of MeSZR compared to controls (in *H. perforatum*, *H. tetrapterum* and *H. calycinum* except M_1). MeSZR had its highest proportion in the total CK pool in the evolutionarily oldest *H. calycinum* and its lowest in the evolutionarily youngest *H. richeri* (in both species except M_1). In the evolutionarily most advanced *H. richeri*, the second most abundant CK riboside was *t*ZR (19–33%) whereas *c*ZR had the lowest proportion (1–2%). In the other three *Hypericum* species, the second most abundant riboside was iPR, followed by *t*ZR. Remarkably, in the evolutionarily oldest *H. calycinum*, tZR was detected as a minor component (1–3%). 

**Figure 10:**
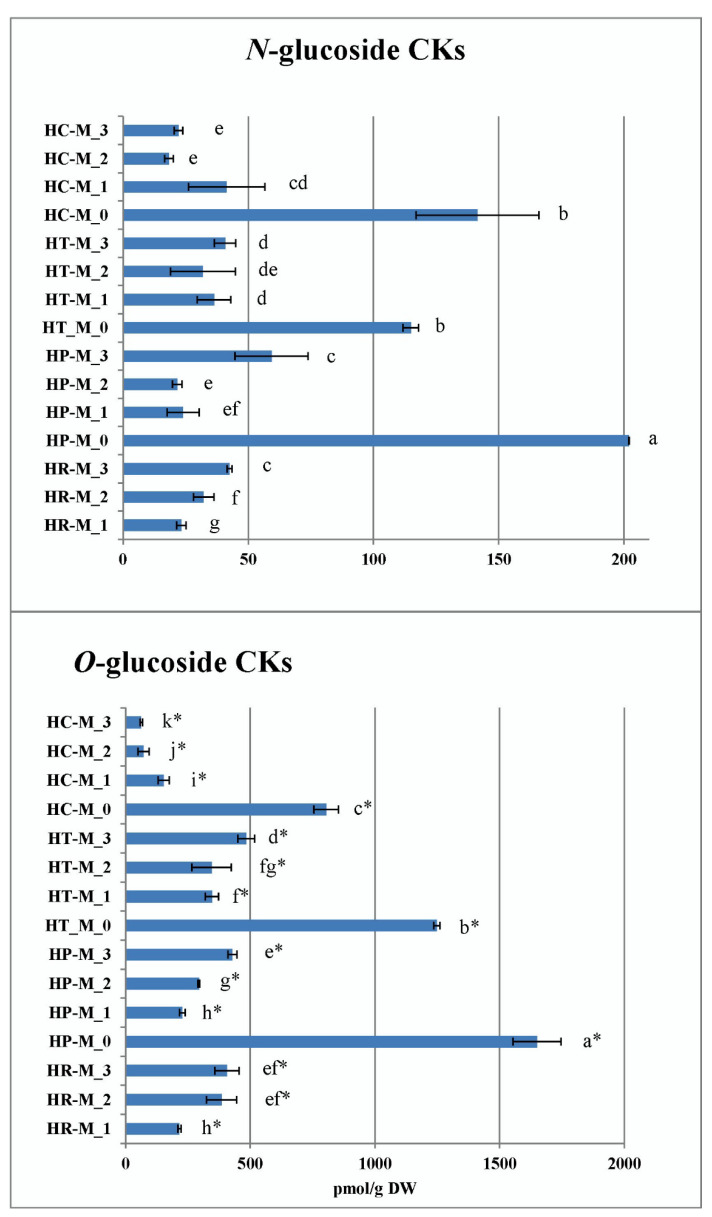
Total sum of cytokinin N- and O-glucosides (upper and lower figure, respectively) in the four *Hypericum* species in M_0—PGR-free, M_1—0.2 mg/L BA, M_2—0.2 mg/L BA + 0.1 mg/L IBA and M_3—0.1 mg/L BA + 0.2 mg/L IBA supplemented media, HR–*H. richeri* Vill., HP–*H. perforatum* L., HT–*H. tetrapterum* Fries. and HC–*H. calycinum*. Same letters indicate statistically non-significant and different letters indicate statistically significant differences in mean values when comparing samples separately for each of the two parameters (comparisons of O-glucoside CKs—distinguished by an asterisk from N-glucosides comparisons).

The pool of endogenous CK *N*-glucosides in *Hypericum* in vitro cultures consisted only of *N9*-glucosides, namely *trans*-zeatin 9-glucoside (*t*Z9G) and dihydrozeatin 9-glucoside (DZ9G), and ranged from 18 pmol/g DW in *H. calycinum* (M_2) to 202 pmol/g DW in *H. perforatum* (M_0). No CK *N7*-glucosides were detected. The levels of *t*Z9G and DZ9G were obviously always higher in the PGR-free control samples (for *H. perforatum*, *H. tetrapterum*, and *H. calycinum*) compared to the PGR-treated variants (Figure 10). Thus, the PGR treatments significantly reduced the pool of CK *N-*glucosides, but no significant general trend was observed as a function of medium composition among the four species. 

Five metabolites contributed to the CK *O*-glucoside pool in *Hypericum* in vitro cultures, namely *trans*-zeatin *O*-glucoside (*t*ZOG) plus its riboside (*t*ZROG), dihydrozeatin riboside 9-glucoside (DZROG) and *cis*-zeatin *O*-glucoside (*c*ZOG) plus its riboside (*c*ZROG). Their content varied from 62 pmol/g DW in *H. calycinum* (M_3) to 1651 pmol/g DW in *H. perforatum* (M_0). When analyzing the data expressing the total sum of CK *O*-glucosides, the highest values for each species were again obtained for the PGR-free control, with *H. perforatum* showing a prevalence ahead of the other species (Figure 10). The evolutionarily oldest *H. calycinum* had significantly lower values in both the PGR-free and PGR-treated samples compared to all three species.

In analogy with our previous data demonstrating total CK pools in *Hypericum* in vitro cultures [24], we clearly indicate here that the evolutionary factors may also affect portions of particular CK metabolic forms such as *c*Z and *t*Z ribosides, methythioderivatives and *O*-glucosides in the *Hypericum* in vitro experimental model. The highest abundance of MeSZR in the total pool of CKs in the evolutionarily oldest *H. calycinum* and the lowest in the evolutionarily youngest *H. richeri* is consistent with the reported biosynthesis of CK methylthioderivatives via the *t*RNA degradation pathway typical of evolutionarily less advanced organisms ([48] and references cited therein). Also, the striking differences in the levels of *c*Z and *t*Z ribosides in *Hypericum* species of different evolutionary ages are consistent with our previous findings that *t*Z-type CKs are mainly found in evolutionarily more advanced plants, whereas *c*Z-types are typical of evolutionarily older cyanobacteria and algae [49], mosses [50], ferns [51] and some less evolved seed plants [52].

#### 2.3.5. Contents of Phenylacetic Acid In Vitro

**Figure 11:**
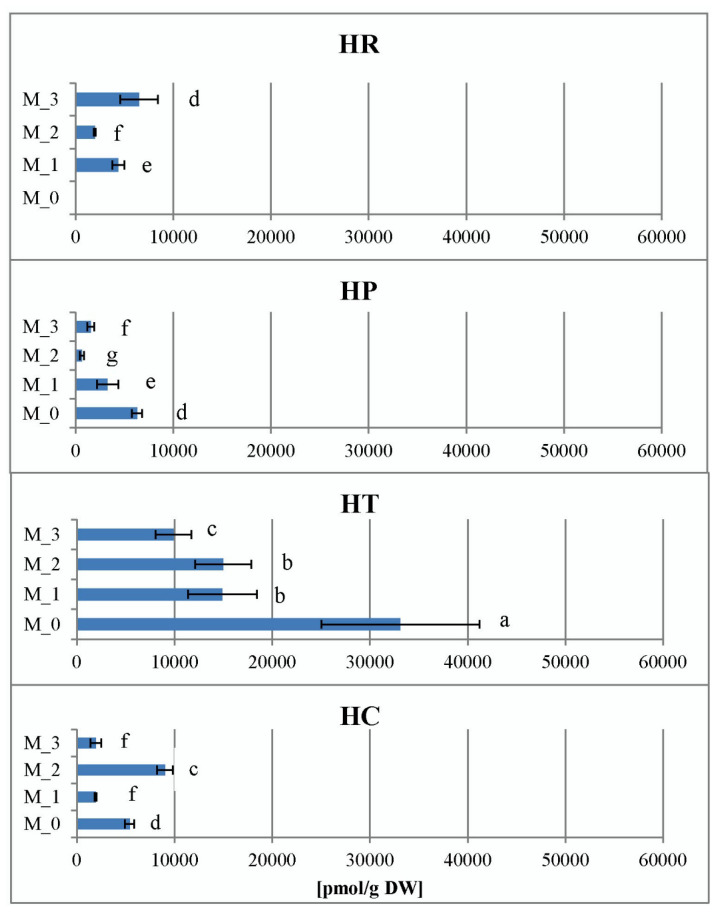
Phenylacetic acid (PAA) levels in the four *Hypericum* species in M_0—PGR-free, M_1—0.2 mg/L BA, M_2—0.2 mg/L BA + 0.1 mg/L IBA and M_3—0.1 mg/L BA + 0.2 mg/L IBA supplemented media. HR–*H. richeri* Vill., HP–*H. perforatum* L., HT–*H. tetrapterum* Fries. and HC–*H. calycinum*. Same letters indicate statistically non-significant and different letters indicate significantly significant differences in mean values when comparing the parameter between all samples.

A non-indole phenolic compound, phenylacetic acid (PAA), was detected in shoot cultures of four *Hypericum* species in vitro at relatively high concentrations, ranging from 640 pmol/g DW in *H. perforatum* (M_2) to 33108 pmol/g DW in *H. tetrapterum* (M_0). When comparing PAA content within species and PGR treatments, *H. tetrapterum* proved to be the species with the highest values of this parameter, whereas no particular trends were evident in the other species/PGR treatment variants (Figure 11). PAA is widely distributed in vascular and non-vascular plants, with higher endogenous levels and lower auxin activity compared with the more widely studied natural indole auxin indole-3-acetic acid (IAA). Therefore, it is thought to play a different role than IAA, as the transport and biosynthesis of PAA occur independently of IAA [53,54].

## 3. Materials and Methods

### 3.1. Plant Material

The plant material for tissue culture of the four *Hypericum* species was collected in the respective floristic regions [55] as follows: *H. richeri* Vill.—its natural habitat in the Vitosha floristic region; *H. perforatum* L. and *H. tetrapterum* Fries.—their natural habitats in the floristic region of the Western Balkan Mountain; *H. calycinum* L.—collected from a cultivation accession in Varna, in the Black Sea coast floristic region. Photographs of the studied representatives of the genus *Hypericum*, belonging to three different sections, can be seen in Figure 1. In addition, for the phytochemical analyses, material of *H. richeri* Vill. was also collected from its natural habitat in the Rila Mountain floristic region.

### 3.2. Tissue Culture Initiation

For shoot culture initiation, the stems of plants growing in situ were cut into 2–3 nodules segments, washed in 70% ethanol for 30 s, sterilized in 0.1% HgCl_2_ for 5 min, and washed in sterile distilled water in triplicate. The sterilized explants were placed in culture medium containing Murashige & Skoog [56] macro- and microelements, Gamborg [57] vitamins, supplemented with 20 g/L sucrose and 0.5 mg/L *N^6^*-benzyladenine (BA), at 25 ± 0.2 °C and 16/8 h photoperiod. After induction of axillary shoot growth, sterile stem explants of 3–4 stem nodules were placed in Murashige and Skoog basic medium, supplemented with 30 g/L sucrose, for long-term maintenance. For *H. richeri*, the addition of 0.2 mg/L BA and 0.1 mg/L indole-3-butyric acid (IBA) to the culture medium was necessary because the plant could not grow and develop in a culture medium without plant growth regulators (PGRs). The subculture period of the stock shoot cultures described in this way was 20 weeks. 

### 3.3. Treatments with Plant Growth Regulators

For the purpose of the tissue culture experiment, CK (BA) and auxin (IBA) were applied exogenously in different combinations as follows: M_0—PGR-free control, M_1—0.2 mg/L BA, M_2—0.2 mg/L BA + 0.1 mg/L IBA and M_3—0.1 mg/L BA + 0.2 mg/L IBA. Media were supplemented with 30 g/L sucrose and 6.5 g/L agar. Plantlets were cultured at 25 ± 0.2 °C with a photoperiod of 16/8 h. Parameters were estimated after 16 weeks of in vitro culture under the described conditions, as plant material was collected from at least five culture vessels. Media were tailored to compare the effect of a single cytokinin treatment (M_1, 0.2 mg/L BA) with a combination of the same treatment with a lower auxin concentration (M_2, 0.2 mg/L BA + 0.1 mg/L IBA) control without plant growth regulators (M_0). In addition, the reversal of the cytokinin–auxin ratio was also tested (M_3, 0.1 mg/L BA + 0.2 mg/L IBA). The selection of the specific plant growth regulators applied (namely benzyladenine and indole-3-butyric acid) was based on our previously reported experiment with genus *Hypericum* [58,59], as well as unpublished experimental in-house laboratory data.

### 3.4. Estimation of Hydricity of the Plant Tissues In Vitro

The fresh biomass of the samples was collected as an average of at least five culture vessels and after obtaining the air dry weight, samples were kept in a desiccator until constant weight was reached. The extent of tissue hydricity was estimated by comparing the ratio of fresh weight to dry weight (FW/DW), as the increase in this parameter characterizes the extent of water accumulation per unit of plant tissue (g). 

### 3.5. Analysis of the Total Amount of Hypericin

#### 3.5.1. Extraction of Plant Material 

The air-dried plant material of the in vitro cultivated and collected from the wild *H. richeri*, *H. perforatum* and *H. tetrapterum* was kept in a desiccator with silica gel as a desiccant before analysis. To obtain the extracts, the ground plant material was initially macerated in chloroform in order to remove chlorophylls and then with methanol in order to obtain hypericin-enriched extract. Maceration with both solvents was at room temperature for 24 h, followed by ultrasonic extraction at room temperature for 15 min in replicate. The extracts obtained were filtered, combined and concentrated in a rotary vacuum evaporator at 40 °C.

#### 3.5.2. Determination of Total Hypericins

The total hypericin content of was determined spectrophotometrically at 588 nm according to the following formula:

*Hyp[mg/g]* = (*A* × *V* × 10)/*m* × 870 (1)

*A = measured absorbance,*

*m = dry weight of the plant material [g],*

*V = volume of the methanol extract [mL],*

870 *= specific absorption coefficient of hypericin [60].*

The measurements were performed in triplicate and the values are given as the mean of three replicates ± standard deviation.

### 3.6. Endogenous Phytohormone Levels Analyses 

Samples of *H. richeri*, *H. perforatum*, *H. tetrapterum* and *H. calycinum* growing in vitro were collected and averaged from at least five culture vessels for each treatment and immediately subjected to freeze-drying. Then, 7–17 mg of the freeze-dried material was subjected to extraction and purification with the addition of isotope-labeled internal standards as previously described [61]. Using reversed-phase and ion-exchange chromatography, two fractions were separated: fraction A—containing hormones of acidic and neutral character (auxins, abscisic acid [ABA], salicylic acid [SA], benzoic acid [BzA], jasmonates and gibberellins), and fraction B—the hormones of basic character (such as CKs). For quantification of endogenous phytohormones, HPLC (Ultimate 3000, Dionex, Sunnyvale, CA, USA) coupled with a hybrid triple quadrupole/Linear ion trap mass spectrometer (3200 Q TRAP, Applied Biosystems, Waltham, MA, USA) was used, as previously described [62,63]. Apart from the gradient programme, the chromatographic conditions for both fractions were the same and included: HPLC column Kinetex C18 (100 × 3 mm, 2.6 μm, Phenomenex, Torrance, CA, USA), flow rate of 0.3 mL/min, solvent A (50 mM acetic acid in water), solvent B (water), and solvent C (95% acetonitrile and 5% water), where solvent A was kept constant at 5%. Linear gradient of 10% to 45% C for fraction A, or 5% to 25%C for fraction B was run in 7 min, followed by 1 min wash at 95% C, and 5 min equilibration to initial gradient conditions. The abbreviations for the CKs were adopted and modified according to Kamínek et al. [64].

### 3.7. Statistical Analyses

Number of repetitions, plant culture vessels, plant individuals, extractions and measurements are given for each respective method. The means were compared using the *t* test of unequal variances at *p* ≤ 0.05. Results are presented as means ± standard errors of the means, unless stated otherwise. Differences were considered statistically significant at *p* ≤ 0.05. In the figures, statistically nonsignificant means were marked with the same letters.

## 4. Conclusions

The relationships between intragenic evolutionary development and the ability to produce hypericin in the genus are a much debated topic in *Hypericum* research. The present study was motivated by the phytopharmacological value of the condensed naphthodianthrones, hypericin and pseudohypericin, and the potential for their biotechnological production by using other *Hypericum* representatives of different evolutionary ages in addition to the widely known *H. perforatum*. It has been shown that the evolutionarily youngest sections of the genus *Hypericum* have the highest potential for hypericin production. The choice of *Hypericum* species of evolutionarily more advanced sections (such as *Drosocarpium*) in the tissue culture protocol could be a prospective tool for enhancing biotechnological delivery of the valuable naphthodianthrones hypericin and pseudohypericin. The distinctive physiological features of *Hypericum* species belonging to different evolutionary stages is crucial to the development of these processes. Thus, for the highest in hypericin potential productivity, *H. richeri*, it was shown that PGRs are a necessary condition to obtain in vitro culture biomass formation. On the contrary, the lowest in hypericin productivity, *H. tetrapterum*, expressed vigorous growth and biomass formation even in media without any PGR treatments. Subtle physiological processes such as tissue water retention, endogenous phytohormone accumulation and conjugation were also shown to interplay with evolutionary level and hypericin productivity of the studied species. The observed relationships between hypericin productivity potential and particular physiological traits suggest that hypericin production may have arisen in the evolutionary development of the genus as an adaptation feature, required by species to adapt to changing conditions in their environment.

## Data Availability

Data is available from the corresponding co-authors upon request.

## Supplementary Materials

The following supporting information can be downloaded at: https://www.mdpi.com/article/10.3390/plants11202753/s1, Figure S1. Proportional representation of abscisic acid (ABA) and its storage conjugate ABA-glucose ester (ABA-GE) in the four *Hypericum* species in M_0—PGR-free, M_1—0.2 mg/L BA, M_2—0.2 mg/L BA + 0.1 mg/L IBA and M_3—0.1 mg/L BA + 0.2 mg/L IBA supplemented media, *HR–H. richeri Vill., HP–H. perforatum L., HT–H. tetrapterum Fries. and HC–H. calycinum;* Figure S2. Proportional representation of salycilic (SA) and benzoic (BA) acids in the four *Hypericum* species in M_0—PGR-free, M_1—0.2 mg/L BA, M_2—0.2 mg/L BA + 0.1 mg/L IBA and M_3—0.1 mg/L BA + 0.2 mg/L IBA supplemented media, *HR–H. richeri Vill., HP–H. perforatum L., HT–H. tetrapterum Fries. and HC–H. calycinum**;* Figure S3. Proportional representation of jasmonic acid (JA) and its bioactive conjugate JA-isoleucine (Ja-Ileu) in the four *Hypericum* species in M_0—PGR-free, M_1—0.2 mg/L BA, M_2—0.2 mg/L BA + 0.1 mg/L IBA and M_3—0.1 mg/L BA + 0.2 mg/L IBA supplemented media, *HR–H. richeri Vill., HP–H. perforatum L., HT–H. tetrapterum Fries. and HC–H. calycinum;* Figure S4. Proportional representation of cytokinin ribosides in the four *Hypericum* species in M_0—PGR-free, M_1—0.2 mg/L BA, M_2—0.2 mg/L BA + 0.1 mg/L IBA and M_3—0.1 mg/L BA + 0.2 mg/L IBA supplemented media, *HR–H. richeri Vill., HP–H. perforatum L., HT–H. tetrapterum Fries. and HC–H. calycinum.*
