# Supplementary material for: Evolutionary Aspects of Hypericin Productivity and Endogenous Phytohormone Pools Evidenced in Hypericum Species In Vitro Culture Model
Source: Plants (Basel). 2022 Oct 18;11(20):2753. doi: 10.3390/plants11202753 (PMC9609395; doi:10.3390/plants11202753)
Supplement: Supplementary file 1 [file plants-11-02753-s001.zip › plants-1922173-Supplementary.pdf]

SUUPLEMENTARY MATERIAL

Evolutionary aspects of hypericin productivity and endogenous phytohormone pools evidenced in *Hypericum* species *in vitro* culture model

Kalina Danova [1,*], Vaclav Motyka [2,*], Antoaneta Trendafilova [1], Petre I. Dobrev [2], Viktorya Ivanova [1] and Ina Aneva

[1] Institute of Organic Chemistry with Centre of Phytochemistry, Bulgarian Academy of Sciences, Acad. Georgi Bonchev Str., bl.9, 1113 Sofia, Bulgaria

[2] Institute of Experimental Botany of the Czech Academy of Sciences, Rozvojová 263, CZ-165 02 Prague 6, Czech Republic

[3] Institute of Biodiversity and Ecosystem Research, Bulgarian Academy of Sciences, 2 Gagarin Str., 1113 Sofia, Bulgaria

* Correspondence: k_danova@abv.bg; Motyka@ueb.cas.cz

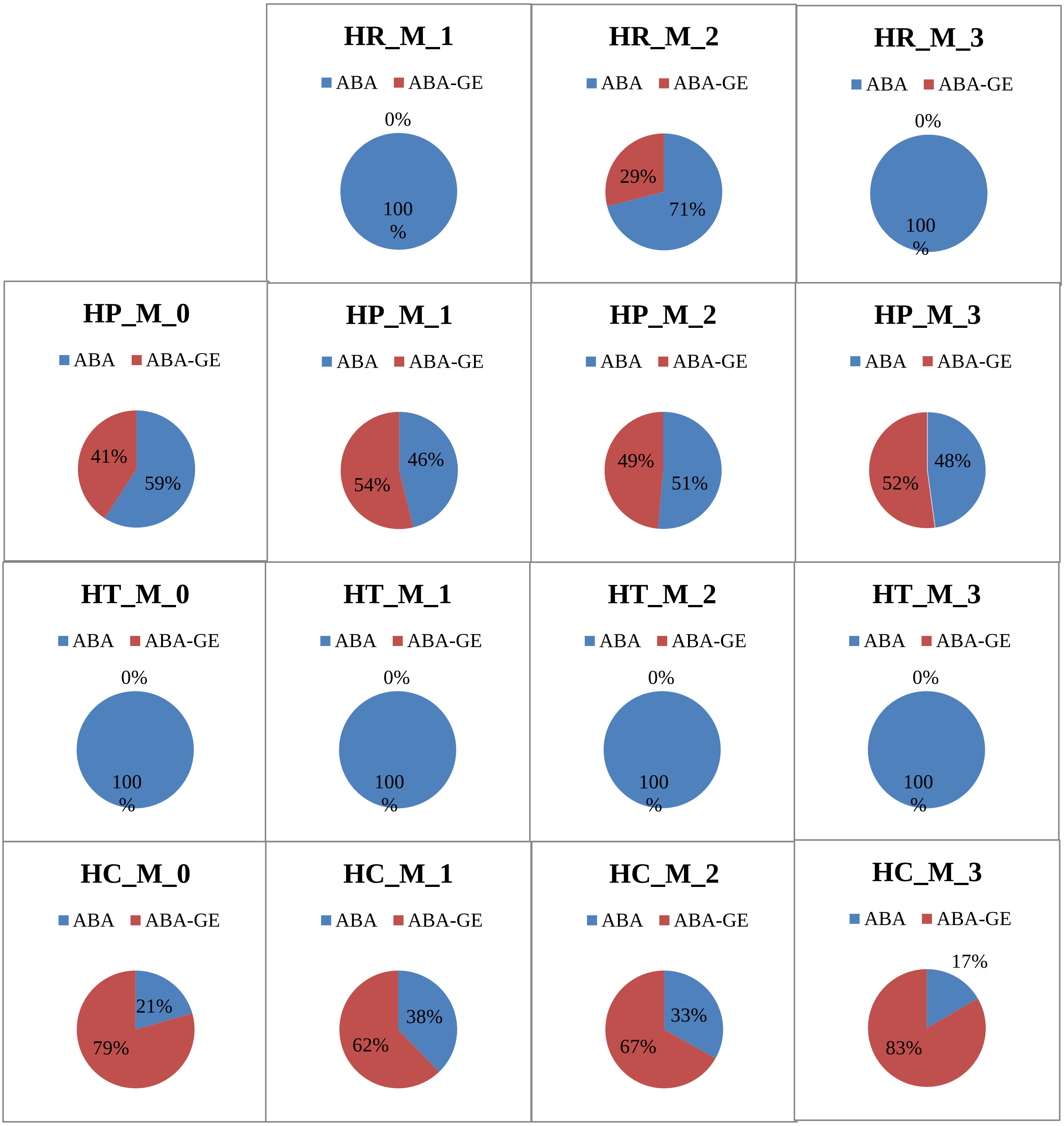

Figure S1. Proportional representation of abscisic acid (ABA) and its storage conjugate ABA-glucose ester (ABA-GE) in the four *Hypericum* species in M_0 - PGR-free, M_1 - 0.2 mg/l BA, M_2 - 0.2 mg/l BA + 0.1 mg/l IBA and M_3 - 0.1 mg/l BA + 0.2 mg/l IBA supplemented media, *HR – H. richeri Vill., HP – H. perforatum L., HT – H. tetrapterum Fries. and HC – H. calycinum.*

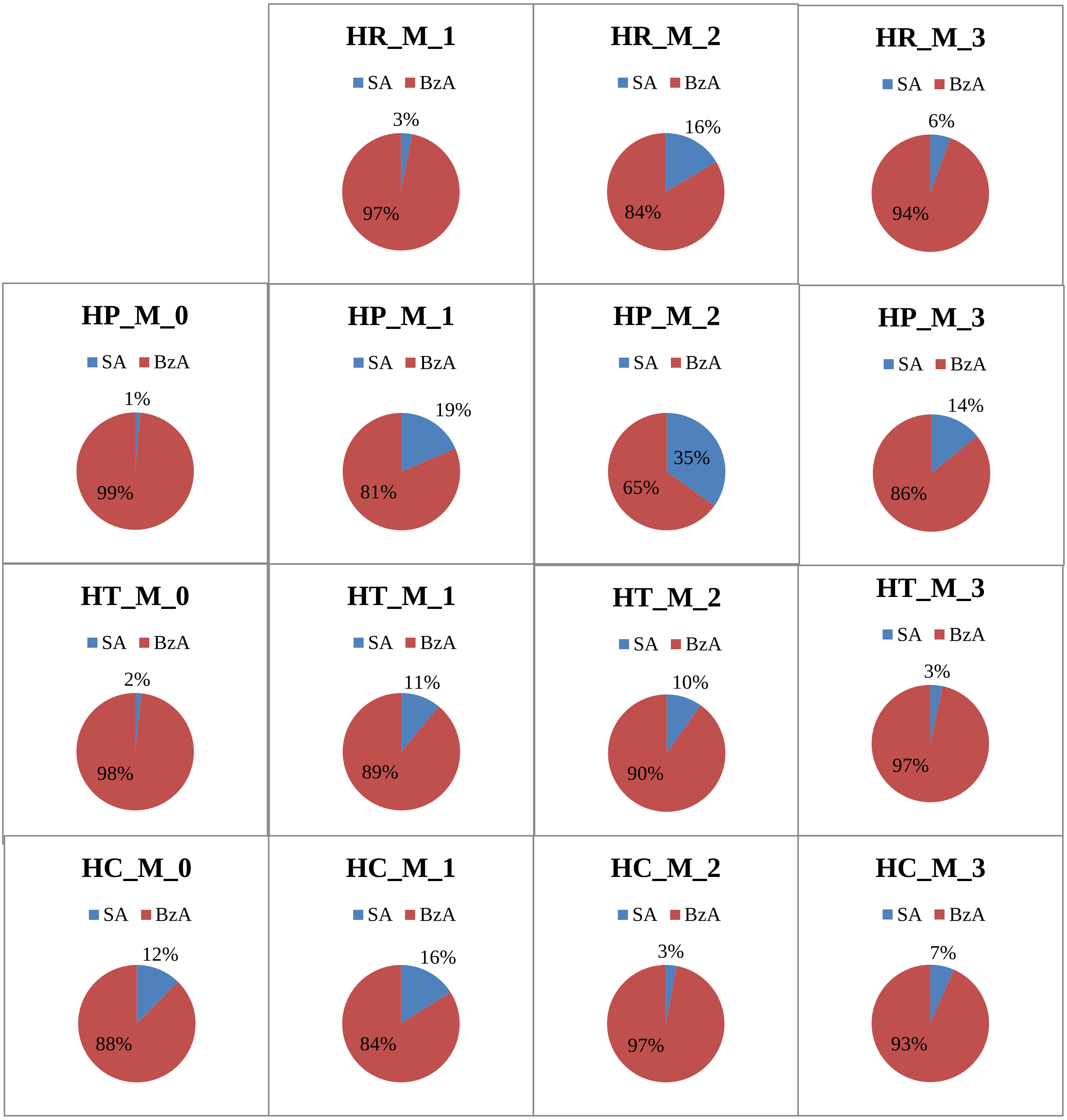

Figure S2. Proportional representation of salycilic (SA) and benzoic (BA) acids in the four *Hypericum* species in M_0 - PGR-free, M_1 - 0.2 mg/l BA, M_2 - 0.2 mg/l BA + 0.1 mg/l IBA and M_3 - 0.1 mg/l BA + 0.2 mg/l IBA supplemented media, *HR – H. richeri Vill., HP – H. perforatum L., HT – H. tetrapterum Fries. and HC – H. calycinum.*

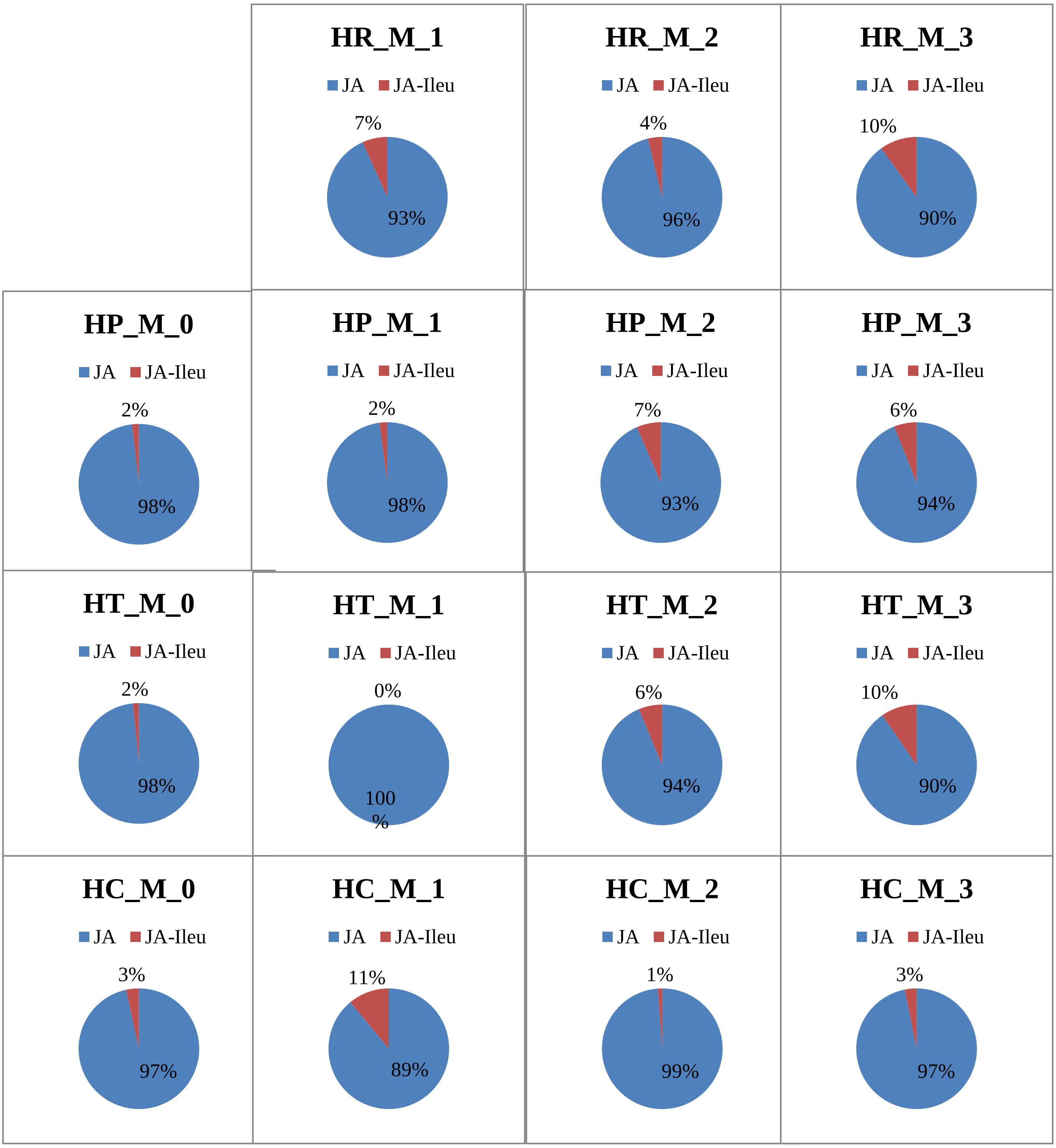

Figure S3. Proportional representation of jasmonic acid (JA) and its bioactive conjugate JA-isoleucine (Ja-Ileu) in the four *Hypericum* species in M_0 - PGR-free, M_1 - 0.2 mg/l BA, M_2 - 0.2 mg/l BA + 0.1 mg/l IBA and M_3 - 0.1 mg/l BA + 0.2 mg/l IBA supplemented media, *HR – H. richeri Vill., HP – H. perforatum L., HT – H. tetrapterum Fries. and HC – H. calycinum.*

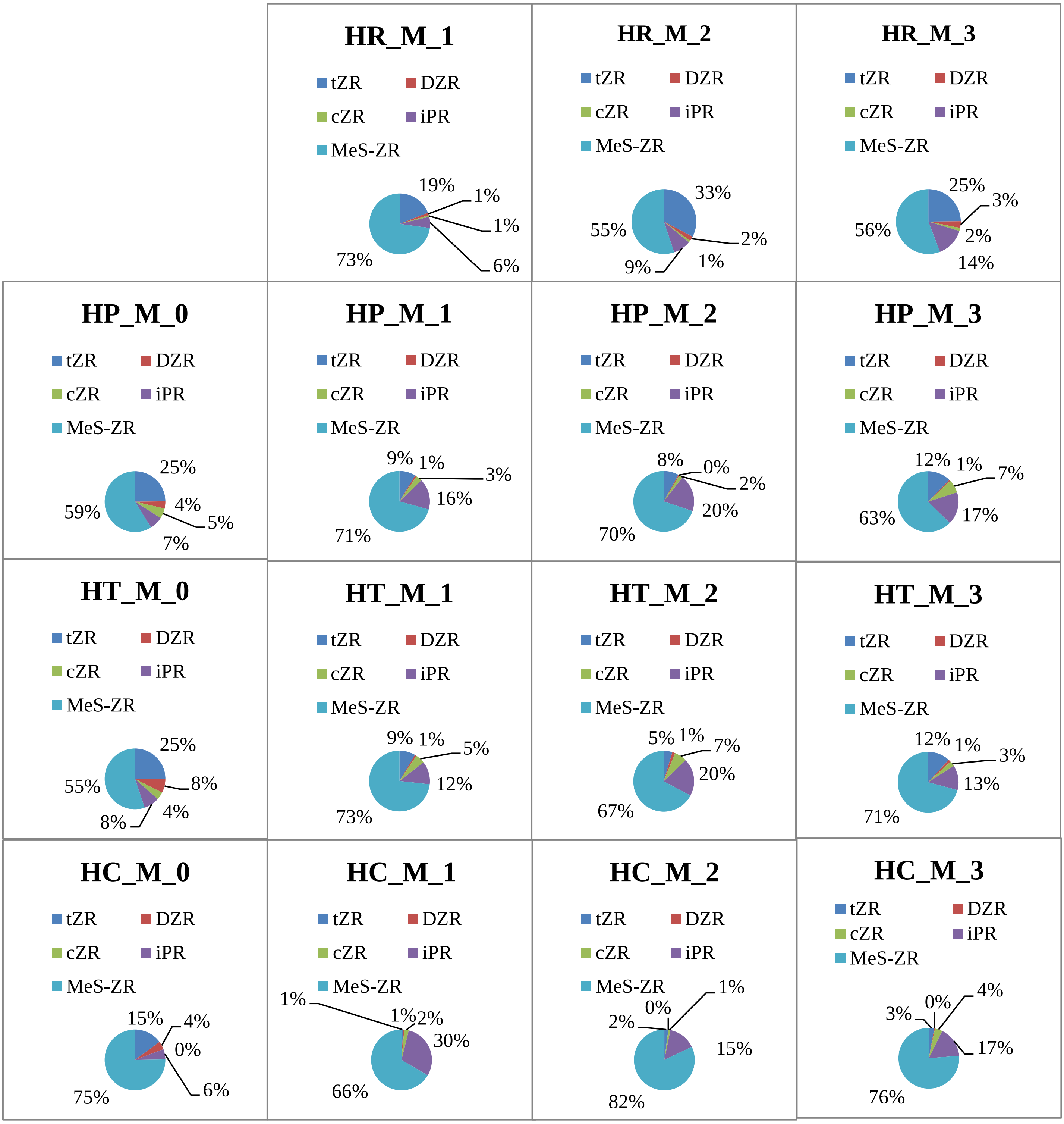

Figure S4. Proportional representation of cytokinin ribosides in the four *Hypericum* species in M_0 - PGR-free, M_1 - 0.2 mg/l BA, M_2 - 0.2 mg/l BA + 0.1 mg/l IBA and M_3 - 0.1 mg/l BA + 0.2 mg/l IBA supplemented media, *HR – H. richeri Vill., HP – H. perforatum L., HT – H. tetrapterum Fries. and HC – H. calycinum.*
